# Chlorophyllin-Based 405 nm Light Photodynamic Improved Fresh-Cut Pakchoi Quality at Postharvest and Inhibited the Formation of Biofilm

**DOI:** 10.3390/foods11162541

**Published:** 2022-08-22

**Authors:** Yuchen Zhang, Zhaoyang Ding, Changbo Shao, Jing Xie

**Affiliations:** 1 College of Food Science and Technology, Shanghai Ocean University, Shanghai 201306, China; 2 National Experimental Teaching Demonstration Center for Food Science and Engineering, Shanghai Ocean University, Shanghai 201306, China; 3 School of Intelligent Manufacturing and Service, Shandong Institute of Commerce and Technology, Jinan 250103, China; 4 Shanghai Professional Technology Service Platform on Cold Chain Equipment Performance and Energy Saving Evaluation, Shanghai 201306, China

**Keywords:** photodynamic inactivation (PDI), antimicrobial, biofilm, fresh-cut pakchoi, preservation

## Abstract

The aim of this study was to evaluate the effect of chlorophyllin-based photodynamic inactivation (Chl-PDI) on biofilm formation and fresh-cut pakchoi quality during storage. Firstly, Chl-based PDI reduced the amount of biofilm in an in vivo experiment and inactivated the food spoilage bacteria. Antibacterial mechanism analysis indicated that the bacterial extracellular polysaccharides and extracellular proteins were vulnerable targets for attacks by the Chl-based PDI. Then, the food spoilage microorganisms (*Pseudomonas reinekei* and *Pseudomonas palleroniana*) were inoculated onto the surface of fresh-cut pakchoi. We used chlorophyllin (1 × 10^−5^ mol/L) and 405 nm light (22.27 J/cm^2^ per day) to investigate the effect of Chl-based PDI treatment on fresh-cut pakchoi quality during storage. The results showed that Chl-based PDI increased the visual quality and the content of chlorophyll, VC, total soluble solids, and SOD activity and decreased the occurrence of leaf yellowing and POD activity. These suggest that Chl-based PDI can be used for the preservation of fresh-cut pakchoi and has the potential to inhibit biofilm formation of food spoilage bacteria. It is of great significance for the effective processing and traditional vegetable preservation.

## 1. Introduction

Nowadays, fresh-cut vegetables are expanding sharply in the global market [1]. However, fresh-cut vegetables are susceptible to contamination by microorganisms during transportation, storage, distribution, and consumption, which results in spoilage and deterioration [1,2]. The Food and Agriculture Organization of the United Nations (FAO) reported that approximately 14% of food loss happens before the retail stage [3]. However, about 9–20% of fruits and vegetables are wasted in the consumption stage [3]. Studies have shown that *Pseudomonas* spp. cause various kinds of fresh-cut vegetables to spoil [4,5,6]. This is evident in cases in which *Pseudomonas* spp. caused lettuce to rot [5] while *Pseudomonas cichorii* resulted in the spoilage of and necrotic spots on heads of lettuce [6]. Another example of *Pseudomonas* spp.-caused spoilage was when pectolytic enzymes produced by *Pseudomonas viridiflava* and *Pseudomonas chlororaphis* accelerated the rate of rot and caused soft rot of vegetables [7,8]. Our previous work found that *Pseudomonas reinekei* and *Pseudomonas palleroniana* showed highly the dominant decaying bacteria of fresh-cut pakchoi [9,10]. Moreover, Morris et al. [11] found that bacteria formed biofilms easily on the surface of vegetables, such as parsley and lettuce, and that the bacterial biofilms formed on the surface of vegetables are difficult to remove, which adversely affects the freshness storage of vegetables. Biofilms enhance bacterial resistance and promote the spread of bacteria, causing great harm to the safety of vegetables [12]. To reduce the microbial contamination, a non-thermal sterilization process, such as UV, sodium hypochlorite, 405 nm light, etc., was used. However, the excessive use of chlorine can be carcinogenic [13] and is forbidden in food industries in Sweden, Germany, Belgium, and other European countries [14]. Additionally, excessive UV exposure is harmful to human skin and eyes and 405 nm light sterilization efficiency is low [10]. Hence, there is a need to develop a new sterilization technology for the fresh-cut vegetable industry.

Plant-based photodynamic inactivation (PDI), which showed high sterilization efficiency, might serve as a promising answer [15,16,17]. PDI is a new, non-thermal processing method used in food and medicine. Chlorophyllin (Chl) is a semi-synthetic porphyrin, water-soluble food colorant, also known as food additive E140 [18]. After being activated by 405 nm light, it produces reactive oxygen species (ROS) around the surface, causing lethal damage to bacteria, viruses, and fungi [19,20]. Studies have shown the potential of PDI in vegetable preservation without a negative impact on quality. This is exemplified in the work that showed that a chlorophyllin photosensitization treatment prolonged the shelf life of strawberries for 3 days and maintained the antioxidant activity and color of strawberries [17]. In a similar case, Paskeviciute et al., (2018) [21] found that Chl-based photosensitization reduced 2.4 log CFU/mL microbiota on tomatoes. Hence, plant-based PDI may open a new way for the development of non-thermal, effective, and eco-friendly antibacterial technology [22]. Additionally, some studies reported that PDI effectively inhibits the formation of biofilm [23,24]. For example, Chen et al., (2020) [24] used curcumin-based PDI eradiated 90% biofilm and reduced the key components of the extracellular polymers of *Vibrio parahaemolyticus*. Another example, in the work undertaken by Silva et al., (2018) [25], showed that rose bengal and erythrosine-based PDI inhibited the biofilm formation of *Staphylococcus aureus* and *Listeria innocua*. Bonifácio et al., (2018) [26] found that curcumin-based, blue-light PDI reduced 4.9 log of viable biofilm of *Listeria innocua.* However, inhibition by Chl-based PDI of biofilm formation on vegetables has been rarely reported.

The aim of this study was to evaluate the antimicrobial efficiency of Chl-based PDI against *Pseudomonas reinekei* and *Pseudomonas palleroniana*. We investigated the effect of Chl-based PDI on the quality of fresh-cut pakchoi during the storage stage by measuring organoleptic properties, color parameters, weight loss, water distribution and migration, soluble solids, chlorophyll content, VC content, superoxide dismutase (SOD) content, and peroxidase (POD) content. Moreover, the inhibition of biofilm by Chl-based PDI was also investigated by confocal laser scanning microscope (CLSM), extracellular polysaccharide, and lipid assays.

## 2. Materials and Methods

### 2.1. LED Lightbox Setup

An LED illumination system was set in a refrigerator (BCD-252MHV, SSEC, Suzhou, China) with high-intensity 405 nm LED lamps (15.12 μmol/(m^2^·s), 5.1 W/m^2^, WAN-T8120, WEGA, Qingzhou, China). The wavelength range of visible light was between 400 and 780 nm. The irradiance of 405 nm LED through the PVC packaging materials at 30 cm (vertical distance) was 5.1 w/m^2^, which was measured by an LED radiometer (ST-513, SENTRY, Taipei, Taiwan).

### 2.2. Culture of Bacterial Strains and the Preparation of Cocktail Solutions

In our previous work, we selected two types of specific spoilage microorganisms (SSOs), *Pseudomonas reinekei* MT1 (*P. reinekei*) and *Pseudomonas palleroniana* (*P. palleroniana*) CFBP4389 [9,10]. *P. reinekei* and *P. palleroniana* were isolated from spoiled pakchoi and caused severe spoilage when they were inoculated with fresh pakchoi. Details of the culture of bacterial strains were described by Zhang et al. [27]. *P. reinekei* and *P. palleroniana* were prepared with a final concentration of approximately 10^6^ CFU/mL.

### 2.3. Chemicals and Treatment Method of Pakchoi

**Figure 1:**
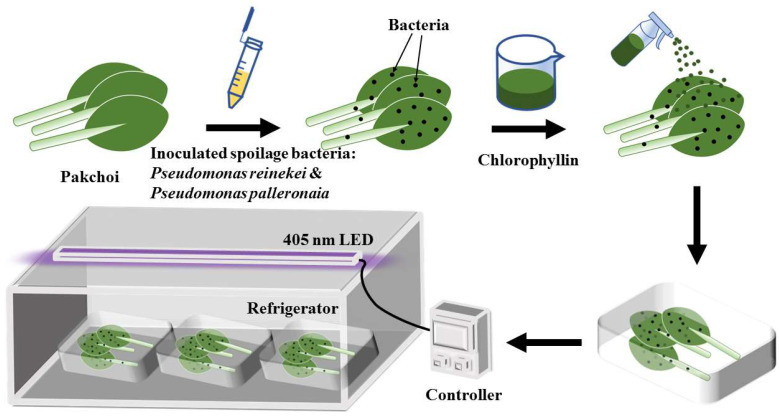
Schematic diagram of the Chl+light group experimental arrangement.

Chlorophyll sodium salt (Chl) was obtained from Yuanye Bio-Technology Co., Ltd., Shanghai, China. The solution was prepared with 1.5 × 10^−5^ M of chlorophyllin. Pakchoi plants were grown on a commercial farm in Pudong, Shanghai, China. Samples were sterilized by 200 ppm of NaClO solution and dried. After that, samples were cut by a sterile scalpel 1 cm away from the root. Then, as Figure 1 shows, the leaves were soaked in the cell suspensions (10^6^ CFU/mL) for 5 min and dried again. The control group was treated without light or Chl. The Chl group was treated with Chl. The light group was treated with light. The Chl+light group was treated with light and Chl. A solution of Chl was sprayed on the surface of leaves of the Chl and Chl+light groups and dried before storage (spray: 5 g of solution per 100 g of sample). Additionally, the same amount of sterile water was sprayed on the leaves of the pakchoi of the light and control groups. Then, the 405 nm LED illumination system provided 12 h of irradiation (22.27 J/cm^2^) every day at 4 °C. Every sample was collected at 0 d, 2 d, 4 d, 6 d, 8 d, 10 d, and 12 d (a total dose of 0, 44.54, 89.08, 133.63, 178.17, 222.71, and 267.25 J/cm^2^, respectively).

### 2.4. Organoleptic Properties, Color Parameters, Chlorophyll Content, and Weight Loss Rate

The methods of organoleptic properties were according to Zhang et al. [9]. The panel was made up of 20 dietetic students and three food science faculty members, who conducted sensory evaluations of pakchoi samples. These students were enrolled in graduate research and experimental foods courses, where they gained experience in translating their perceptions of color, smell, and form into descriptive words and numbers on scoring sheets. The panel that conducted a sensory evaluation of samples used a 10-grade marking system: 10.0–9.0 (excellent), 8.9–7.0 (good), 6.9–5.0 (fair), and 4.9–0 (discardable). The surface color of pakchoi was determined using a CR-400 Chroma Meter (Konica Minolta Sensing Inc., Osaka, Japan). In order to have homogeneous color samples, the measurements used were only obtained from the green part of the pakchoi leaf. The color coordinates ranged from L* = 0 (black) to L* = 100 (white), −a* (greenness) to +a* (redness), and –b* (blueness) to +b* (yellowness).

The methods of determining the chlorophyll content were according to Hasperué et al. [28] with some modifications. A total of 5 g of leaf tissue of pakchoi was homogenized in 20 mL of 80% acetone with a tissue homogenizer at 2000× g for 30 s. The absorbance of the filtered homogenate was measured in UV-1102 (Tianmei Instrument Co., Ltd., Changsha, China) at 645 and 663 nm. The weight loss (%) was calculated using the following equation:

(1) $$X=\frac{W_{0}-W_{1}}{W_{0}}\times 100\%$$

where X = weight loss rate (%), W_0_ = the weight of pakchoi on day 0 in g, and W_1_ = the weight of pakchoi with different treatments in g.

### 2.5. Soluble Solids, Water Distribution and Migration, VC Content, Superoxide Dismutase (SOD), and Oxidase (POD) Content

The methods of determining the soluble solids and water distribution and migration content were according to Zhang et al., (2021) [9]. We took 5 g of a sample and fully grinded and centrifugated it for 10 min at 3500 rpm. Then, we took a drop of the supernatant on the inspection mirror of a digital refractometer (PR32a, ATAGO, Japan) and recorded the reading. The pakchoi samples were cut into 1 cm × 1 cm pieces and tested by LF-NMR (0.5 T, 23.2 MHz, PQ001, Niumag Electric Co., Shanghai, China) for water distribution and migration testing. Acquisition parameters were as follows: coil temperature = 32 °C, proton resonance frequency = 24 MHz, CPMG sequence was used, SW (sampling frequency) = 100 Hz, RG_1_ (analog gain) = 20, P_1_ = 20.00 μs, DRG1 (digital gain) = 3, TD = 1024, PRG1 = 3, TW (repeated sampling times) = 15,000, NS (accumulation times) = 4, P = 35 μs, TE (echo time) = 0.500, and echo number (nech) = 3000. The T_2_ spectrum was obtained by iterative inversion using the analysis software provided by Niumag Electric Co., Ltd., Shanghai, China. The methods of determining the VC content were according to Wang et al. [29]. The leaf tissue (1.0 g) of samples was ground in 5 mL 0.05 mol L^−1^ oxalic acid-0.2 mM EDTA. The supernatant was collected by centrifuging at 13,000× *g* for 20 min. The methods of determining the SOD and POD activity were according to He et al. [30]. The reaction mixture consisted of a supernatant (0.5 mL), 0.1 mol L^−1^ PBS (1 mL, pH 7.8), 0.2 % (*v*/*v*, 0.9 mL) guaiacol, and 0.3% (*v*/*v*, 0.6 mL) H_2_O_2_.

### 2.6. Pseudomonas spp. Count, Extracellular Polysaccharide, Extracellular Protein, and Attached Biomass

The *Pseudomonas* spp. count was determined according to Federico et al. [5]. We weighed 10 g of fresh-cut pakchoi samples in the aseptic operation platform, then put 90 mL 0.1 M PBS into an aseptic homogenizing bag, and made a 1:10 sample homogenization solution. The CFC (Qingdao Haibo Biotechnology Co., Ltd., Qingdao, China) was used to culture and determine *Pseudomonas* spp. count.

Sterile stainless steel sheets were added to the LB medium supplemented with bacteria; then, light (22.27 J/cm^2^ per day) or Chl (1.5 × 10^−5^ M) treatment was used. The no light and Chl treatment group was used as the control. Biofilm extracellular polysaccharides and proteins were measured after a standing culture for 0 d, 6 d, and 12 d at 4 ℃. The biofilm was peeled off into sterile water by ultrasound; then, pH was adjusted to neutral. The extracellular polysaccharides’ content and extracellular protein were measured by the phenol-sulfuric acid method and coomassie brilliant blue method, respectively [31]. The biofilm biomass was quantified using crystal violet staining [32].

### 2.7. Confocal Laser Scanning Microscope (CLSM)

Samples were washed with deionized water to remove unattached bacteria. Then, the samples were stained with SYBR Green I fluorescence. A CLMS machine (LSM710, Carl Zeiss, Oberkochen, Germany) was used to monitor samples that excited at 488 nm and emitted at 500–550 nm [24].

### 2.8. Data Analysis Method

All experiments were performed for at least three independent trials. All data were expressed as mean ± standard error (*n* = 3) and performed by one-way analysis of variance (ANOVA). Additionally, the differences among the means were compared by Duncan’s multiple range test with a significance of *p* < 0.05 using the SPSS 22.0 statistical program.

## 3. Results and Discussion

### 3.1. Inhibition of Chl-Based PDI against Inoculated Spoilage Microorganisms

**Figure 2:**
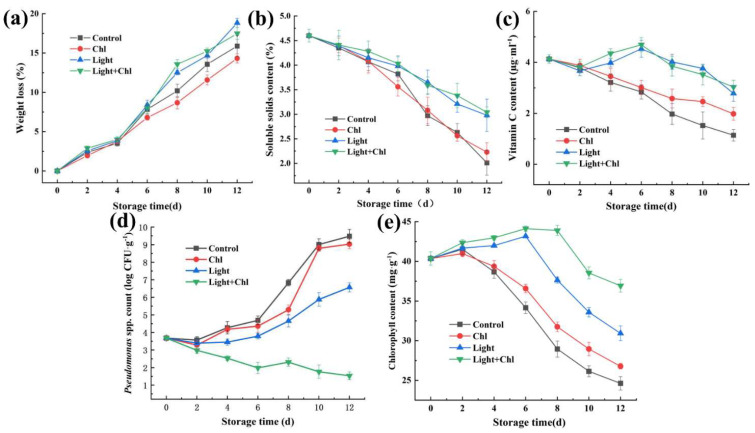
Changes in weight loss (**a**), soluble solids’ content (**b**), chlorophyll content (**c**), vitamin C content (**d**), and *Pseudomonas* spp. count (**e**) of samples with different treatments. Error bars represent the mean ± SE (*n* = 3).

**Figure 3:**
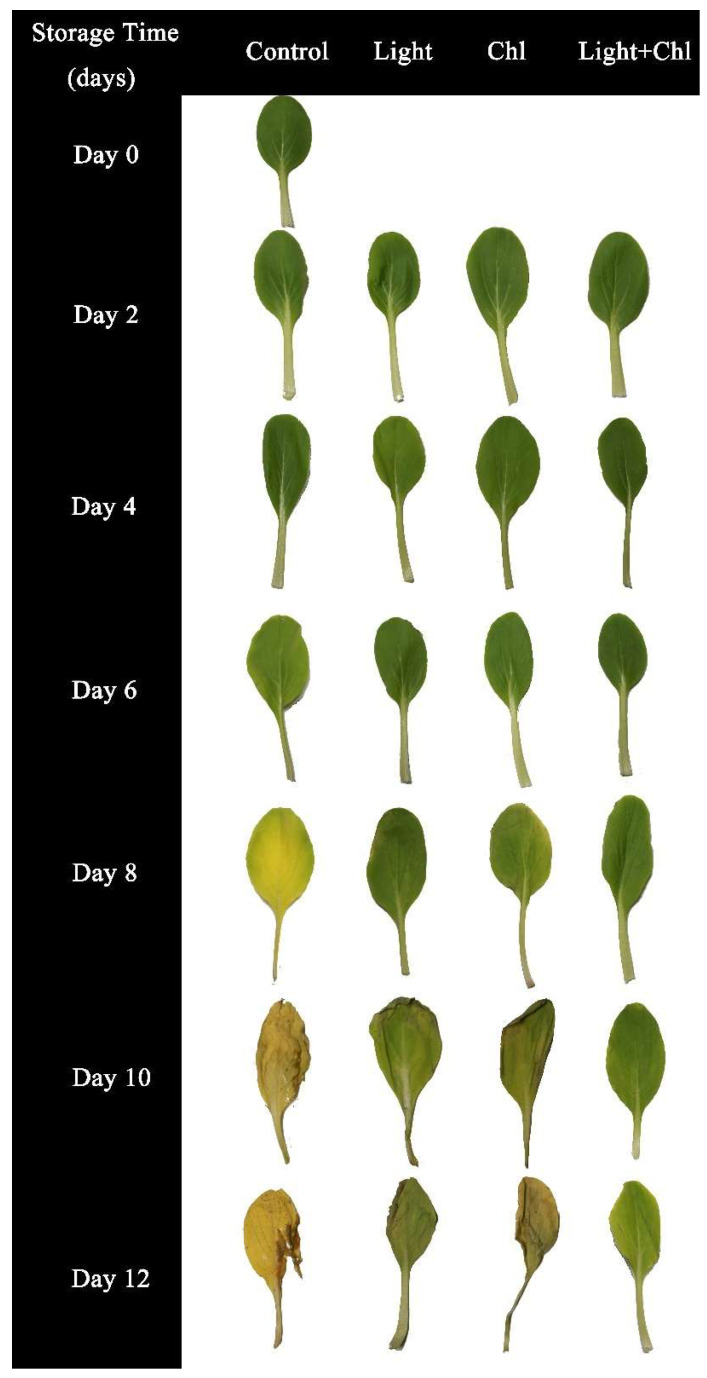
Change of samples in different groups during storage.

In the present study, the inhibitory effect of Chl-based PDI on inoculated spoilage microorganisms was investigated with different treatments (Figure 2d). The initial populations of colonies on samples were 3.68 ± 0.15 log CFU/g after inoculation. However, the bacteria in the control and Chl groups increased rapidly from day 4. By day 10, the populations of microorganisms in the control and Chl groups reached to 6.83 ± 0.18 log CFU/g and 5.29 ± 0.28 log CFU/g, respectively. As Figure 3 shows, the samples at day 10 were already rotten, which mainly resulted in the destruction of plant tissues by the multiplication of spoilage microorganisms. Data obtained in our previous study showed that 405 nm light treatment effectively inactivates bacteria from *P. reinekei* and *P. palleroniana* [10]. These results urged us to further investigate the photodynamic inactivation effect and the actual preservation effect of Chl-based PDI. In this experiment, data showed that the populations of colonies in the light group and Chl+light group were significantly controlled (*p* < 0.05). The populations of colonies in the Chl and Chl+light groups decreased by 13.91% and 74.23%, respectively, compared to the control group by day 10. Compared to light sterilization alone, 70.07% more bacteria were inactivated with the addition of Chl, which significantly improved the efficiency of sterilization by 405 nm light. These data are consistent with the studies by Buchovec et al., (2017) [22] and Luksiene et al., (2019) [17]. This overall reduction in microbial contamination by Chl-based PDI treatment extended the disease-free period of the samples by 4 days (Figure 2d and Figure 3). Therefore, Chl-based PDI treatment, which greatly enhances the efficiency of visible light inhibition, could effectively inhibit the reproduction of microorganisms and control the populations of microorganisms in quantity.

### 3.2. Anti-Biofilm Effects of Chl-Based PDI against Inoculated Spoilage Microorganisms

**Figure 4:**
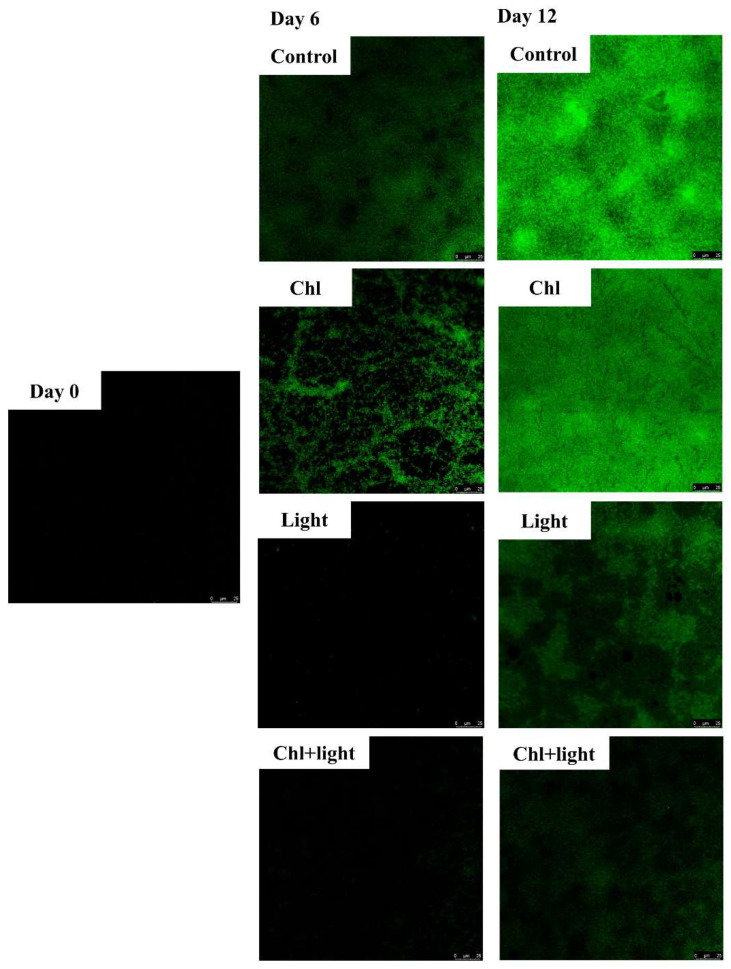
The figures of biofilms’ formation on samples by using CLSM.

**Figure 5:**
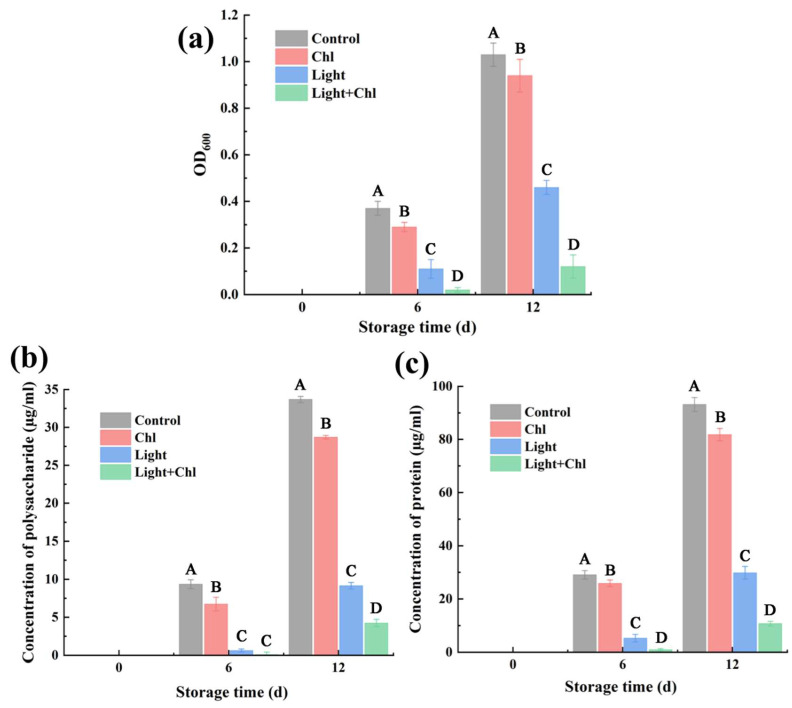
The biofilms’ formation on samples (**a**). The effect of Chl-based PDI on extracellular polysaccharides’ concentration (**b**) and extracellular protein concentration (**c**). ^A, B, C, and D^ mean significantly different in groups (*p* < 0.05). Error bars represent the mean ± SE (*n* = 3).

Biofilms contribute to the persistence of microbial contamination by shielding pathogens and spoilage bacteria from environmental stresses, acting as hot spots for horizontal gene transfer (HGT) of virulence genes, transforming previously benign strains into pathogens [33], and providing niches for antimicrobial-resistant mutagenic activities [34]. Thus, biofilms pose great potential hazards for food safety and human health, which prompted the development of the PDI technique. The anti-biofilm effect of the Chl-based PDI treatment was observed by using CLSM (Figure 4). On day 0, no biofilm was produced. However, at day 6, a small amount of biofilm appeared in both the Chl and control groups, while little biofilm was observed in the light and Chl+light groups (Figure 4—Day 6). Figure 5a shows the change of OD_600_ using crystal violet staining, which quantified the biofilm content. On day 6, compared to the control group, the amount of biofilm was reduced by 70.27% and 94.59% in the light and Chl+light groups, respectively (*p* < 0.05). On day 12, although the OD_600_ of the control group reached 1.03, the amount of biofilm of the light and Chl + light groups decreased by 55.34% and 88.35%, respectively (*p* < 0.05). This indicated that Chl-based PDI or light treatment effectively inhibited biofilm production during storage. The same results were found in studies on chili peppers [35], whole milk [36], salmon [37], and citrus fruits [38].

**Figure 6:**
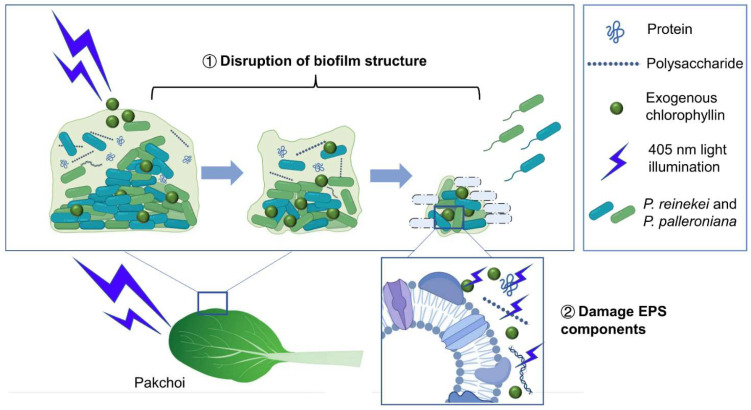
Schematic illustration of biofilm destruction by Chl-based PDI. Chl-based PDI disrupted biofilms by disrupting biofilm structure and subsequent dispersion/reduction in attached microorganisms as well as by disrupting extracellular polymeric substance (EPS), including extracellular proteins and polysaccharides.

As shown in Figure 5b, the concentration of extracellular polysaccharides decreased by 72.84% and 87.41% after light or Chl+light treatment for 12 days, respectively (*p* < 0.05), while the concentration of extracellular protein decreased by 67.98% and 88.46%, respectively (*p* < 0.05, Figure 5c). Extracellular polymeric substance (EPS) consists mainly of substances such as extracellular proteins and extracellular polysaccharides. However, EPS secretion contributes to bacterial colonization or proliferation [39]. Obviously, Chl-based PDI significantly inhibited the secretion of extracellular polysaccharides and extracellular proteins, and the scavenging effect was significantly higher. These speculations were further validated by CLSM observations, which elucidated the effect of Chl-based PDI on the removal of biofilm. The infection of spoilage bacteria is a complex process involving factors such as colonization, proliferation, and biofilm formation. Colonization is achieved by specific appendages or by the production of EPS. In the present experiment, Chl is based on the type II PDI mechanism, through the generation of singly linear oxygen by coupling the excited state photosensitizer to the ground state oxygen, thereby killing cells [40]. In addition, the Chl-based PDI disrupted biofilms by disrupting biofilm structure and subsequent dispersion/reduction in attached microorganisms as well as by disrupting extracellular polymeric substance (EPS), including extracellular proteins and polysaccharides (Figure 6). This opens up the application of Chl-based PDI in addressing food safety associated with bacterial biofilms.

### 3.3. Changes in Weight Loss and Water Distribution

During the storage stage, the increase in weight loss was caused by the water loss [41]. Since light causes the opening of the stomata of the leaves, light treatment might cause an increase in weight loss during storage [42]. We tested to learn if the light dose used in the present study negatively affected the samples. Figure 2a reveals that there was a gradual increase in weight loss of all groups during storage. From day 0 to day 12 of storage, the differences in weight loss were insignificant for all groups. However, at the beginning of day 8, the weight loss of the light and Chl+light groups was slightly higher than that of the control and Chl groups. This indicated that the dose of 22.27 J/cm^2^ per day did not have a significant negative effect on the sample’s weight loss.

**Figure 7:**
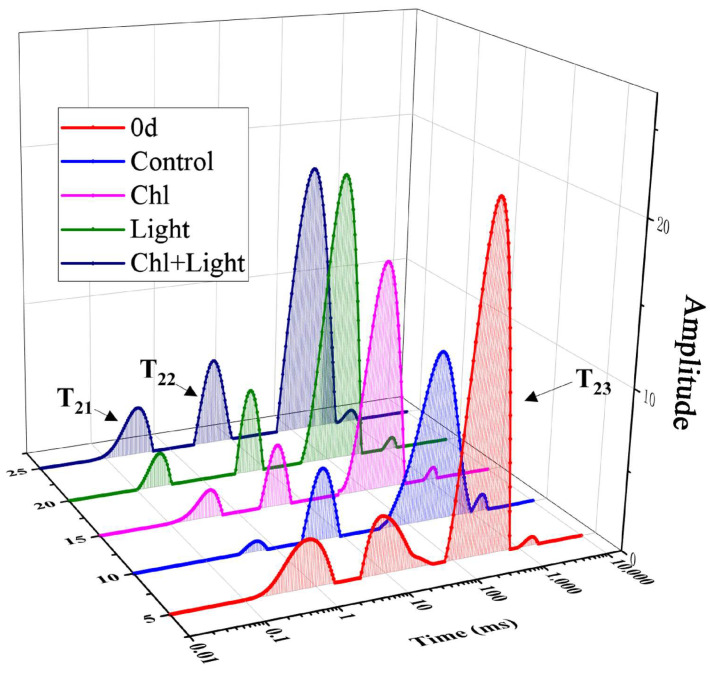
Changes in water distribution of samples with different treatments.

Low-field NMR analysis helped us to better understand the moisture composition in the samples. Figure 7 shows the day 0 and day 12 water distribution of different samples. There were three peaks, representing the three relaxation components, called T21 (<2 ms), T22 (2–20 ms), and T23 (>1000 ms), which represent bound water, immobilized water, and free water, respectively (Figure 7) [43]. After 12 days of storage, the light and Chl+light treated groups’ T23 peak decreased, which indicated that part of the free water had been lost. This was mainly caused by the transpiration and respiration of the leaves. When the steam pressure differed between the water in the blade and that generated outside, the water would dissipate through the pores [44]. The opening of the stomata accelerates this. Meanwhile, both the light and Chl+light groups had higher peak T22 and T21 than the other two groups, which may be due to the 405 nm light triggering the active internal biochemical reactions and accumulation of carbohydrates and sugars of the leaves [45]. Furthermore, the external environment such as the bacterial reproduction and low temperature will cause the adverse reactions, which lead to the participation of a large amount of water. Therefore, we speculated that the higher peak of T22 and T21 than the other two groups might have been due to the light and Chl-based photodynamic treatment alleviating the environmental stress during vegetable storage.

### 3.4. Changes in Total Soluble Solids (TSS) Content and Ascorbic Acid (AsA) Content

TSS in leafy vegetables includes soluble sugar, vitamins, and other water-soluble substances [42]. The initial TSS of the samples was 4.60 ± 0.13. Figure 2b shows that there was a gradual decrease in the content of TSS. However, the Chl and control groups had a sharply decreased TSS from day 8, which was significantly lower than the other groups (*p* < 0.05). The high TSS depletion observed in the Chl and control groups might have been caused by microbial spoilage or a higher energy consumption [46], while the other two groups, which were treated with light, showed a smaller decrease in TSS content. This was due to the activation of photosynthesis by a light note and the degradation of insoluble starch and other substances in the plants into soluble sugars, increasing the content of soluble sugars and other substances in the plants and supplementing the energy consumption of sugars and other substances in the leafy vegetables after harvest, thus maintaining the stability of the soluble solids’ content. The decrease in TSS content of vegetables during refrigeration is mainly due to the depletion of energy metabolism, while microbial colonization accelerated the decrease in TSS. It has been suggested that, compared with the effect on vegetable tissue respiratory stimulation, light treatment has a greater effect on microbial inactivation [46]. Furthermore, TSS can help vegetables overcome low-temperature stress and improve freshness quality [47].

AsA is an important antioxidant substance in vegetables; it also affected the degree of vegetable browning. The initial AsA content of the samples was 4.13 ± 0.17 µg·mL^−1^. However, Figure 2d shows that the AsA content of different groups showed different trends. The ASA levels in both the light and Chl+light groups showed a trend of increasing and then decreasing, and reached a peak on day 6 (4.53 ± 0.33 and 4.69 ± 0.28 µg·mL^−1^, respectively). Since the ASA content of the control and Chl groups gradually decreased, it was significantly lower than that of other two groups on day 6 (2.84 ± 0.28 and 3.01 ± 0.28 µg·mL^−1^, respectively). The reason for this difference was the accumulation induced by light-activated respiration and photosynthesis [48]. Witkowska et al., (2013) proved that AsA content and soluble carbohydrate content in fresh-cut lettuce through light treatment during storage also improved the appearance of vegetables and extended their shelf life [49]. However, as the storage time went on, the rate of the generated AsA could not keep up with the accumulation rate of oxidized substances caused by aging [50]. Therefore, the content of the light and Chl+light groups began to gradually decreased from day 6 and finally went down to 2.78 ± 0.31 and 3.03 ± 0.27 µg·mL^−1^ on day 12, respectively, still significantly higher than that of the control and Chl groups (1.14 ± 0.23 and 1.98 ± 0.25 µg·mL^−1^, respectively, *p* < 0.05). These results are consistent with the finding of Tao et al., (2019) [46] and Witkowska et al., (2013) [48], that light causes a trend of increasing and then gradually decreasing AsA content during storage.

### 3.5. Changes in Chlorophyll Content, Color, and Organoleptic Properties

Chlorophyll content was an important index to evaluate the senescence and commercial value of leafy vegetables. Chlorophyll, a tetrapyrrole compound containing magnesium ions, easily replaced magnesium ions with hydrogen ions in dark conditions during storage, causing plant leaves to brown and yellow [45]. The initial content of chlorophyll was 40.36 ± 0.85 mg·g^−1^. The chlorophyll content showed a trend of increasing and then decreasing. However, the chlorophyll content of the control group and the Chl-only group increased up to day 2, 41.53 ± 0.67, 40.98 ± 0.4 mg·g^−1^, respectively. After that, the chlorophyll content gradually decreased and finally went down to 24.62 ± 0.84 and 26.77 ± 0.35 mg·g^−1^ at day 12, respectively. It was shown that the decrease in the chlorophyll content during storage might have been due to the rupture of the intracellular membrane, resulting in the degradation of chlorophyll by the action of chlorophyllase. However, the light treatment delayed the occurrence of the turning point of the change of chlorophyll content, in which the content of the light and Chl+light groups increased until day 6 (43.17 ± 0.15, 44.13 ± 0.33 mg·g^−1^, respectively). The chlorophyll content then gradually decreased until day 12, when it went down to 30.92 ± 0.93, 36.92 ± 0.8 mg·g^−1^, respectively. From day 4 until the end of storage, the chlorophyll content of the light and Chl+light groups was significantly higher than that of the other two groups (*p* < 0.05). Chlorophyll loss in leafy vegetables, which leads to leaf yellowing or browning, usually implies senescence. However, the low intensity of the LED light increased chlorophyll production and delayed senescence during storage [51,52,53,54]. Therefore, the low dose of light treatment in the present experiment effectively enhanced the chlorophyll content.

Color is one of the main sensory characteristics of fresh vegetables and determines the consumer acceptance of agricultural products. Figure 3 shows photos of different samples during storage. Table 1 shows the changes in L* of the samples. The larger L* value, the brighter the color was; the opposite represents a darker color. The L* of the control group showed a trend of increasing and then decreasing. Combined with the results from Figure 3, it can be seen that the fresh samples showed a dark green color and presented a lower L* value; as the storage time went on and the degradation of chlorophyll occurred, the color of the samples gradually became lighter and the L* value gradually increased. At the end of storage, the L* value decreased again due to the severe browning of the control group, which showed a yellowish-brown color. The same trend occurred in the Chl group that lacked light treatment. However, the L* value of the light and Chl+light groups had a smaller fluctuation range, which indicated that the light treatment could keep the color of the samples in a more stable range.

Table 2 and Table 3 show the a* and b* values of each group during storage, respectively. The a* represents a color bias towards red (+) or green (−), and b* represents a color bias towards yellow (+) or blue (−). The initial value of a* was −17.99 ± 2.76, and the fresh samples appeared dark green. However, the a* values of the samples from the control group gradually increased during the storage, which indicated that the color of the samples increasingly took on a yellow hue. From day 8, the a* value of the control group (−7.85 ± 3.78) was significantly higher than that of the previous 6 days and higher than that of the other three groups. This indicated that the samples in the control group started to yellow severely on day 8 and presented more severely than all the other groups. The b* value of the control group increased and then decreased, which was caused by the leaves turning yellow in the middle stage of storage and gradually getting brown under the action of microorganisms in the later stage. This is consistent with the results found in other studies, that microorganisms cause accelerated browning of leaves [6,7,8]. However, the yellowing of the leaves was better inhibited by the light and Chl+light groups. The light group showed significant changes only from day 10 (*p* < 0.05), while the Chl+light group also showed no significant changes in a* values during the storage. These indicated that light significantly inhibits yellowing or microbially induced browning of vegetables during storage. Similar results were found for LED treatment of fresh-cut lettuce [46], purple kale [55], and baby mustard [56] during storage.

**Figure 8:**
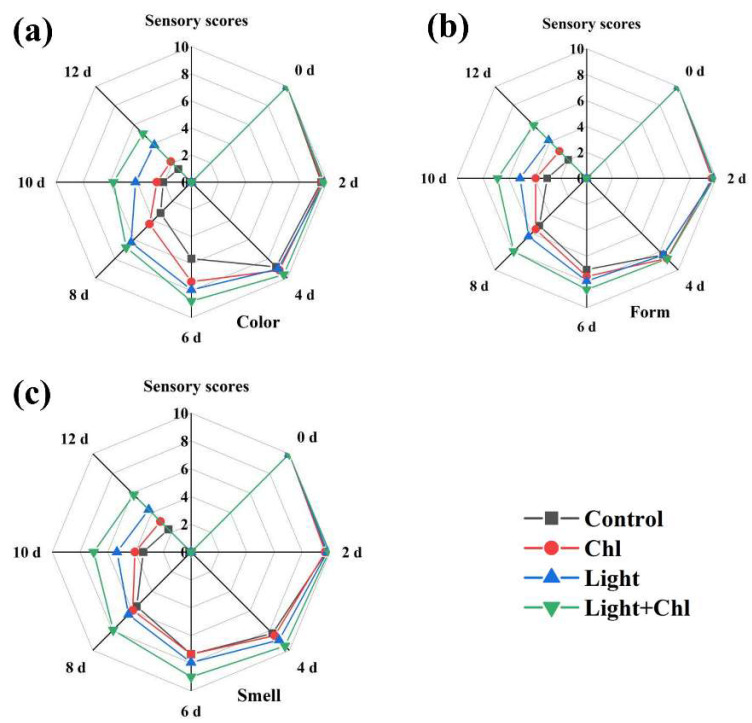
Changes in color (**a**), form (**b**) and smell (**c**) of organoleptic properties’ results in different groups.

To determine the effect of the Chl-based PDI treatment on the samples, a panel of 23 judges rated the samples according to their senses. According to the results obtained (Figure 8), the Chl+light group achieved better sensory scores in terms of color, form, and smell. Additionally, the judging panel did not experience any off-flavors of the Chl-based PDI treatment samples. These results showed that Chl-based PDI had no negative effect on the samples. However, on the 10th day and 12th day, the control group was spoiled with off-odor and browning, which were regarded as unacceptable by the panelists. Combined with the form analysis, the low grade of organoleptic properties of the control group was mainly due to the peculiar smell, leaf yellowing, and wilting.

### 3.6. Changes in Antioxidant Enzyme Activity

**Figure 9:**
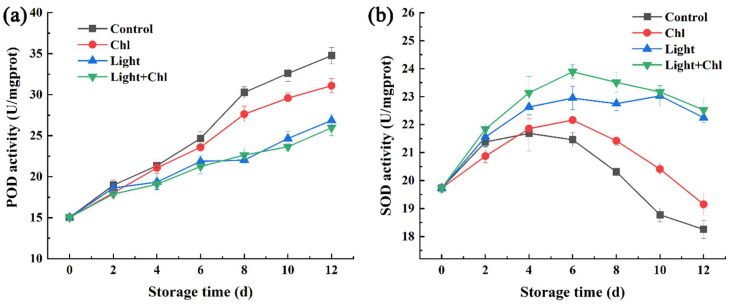
Changes in POD (**a**) and SOD (**b**) activity of samples. Error bars represent the mean ± SE (*n* = 3).

During storage, the content of reactive oxygen species (ROS) increases with the aging of leaves [57]. Meanwhile, the accumulation of ROS, such as H_2_O_2_ and O^2−^ causes oxidation stress or oxidation damage to cells [58]. SOD, the main antioxidant enzyme in vegetables, plays an important part in eliminating excessive ROS in vegetables [59]. Therefore, it is necessary to investigate whether changes in antioxidant activity occurred after photosensitization treatment. Figure 9 shows the changes in POD and SOD activity. The POD activity of each group gradually increased during storage (Figure 9a). From day 6, the activity of the control or Chl groups was significantly higher than that of the two groups with light treatment (*p* < 0.05). This indicated that light effectively inhibited the increase in POD activity. The increase in POD activity often leads to the browning of vegetables [60]. Therefore, light treatment had the potential to reduce vegetable browning by inhibiting POD activity. The SOD content showed a trend of increasing and then decreasing (Figure 9b). From day 4 onwards, the two groups with light treatment showed significantly higher SOD activity than the other two groups. In fact, plant cells usually tightly control ROS levels by producing or activating antioxidant enzymes. This suggested that light treatment or photodynamic treatment helped to enhance antioxidant enzyme activity or reduce ROS levels in vegetables during storage. Similar results were found in pakchoi [45] and fresh-cut celery [50].

## 4. Conclusions

In conclusion, Chl-based PDI effectively improved pakchoi quality at postharvest and inhibited the formation of biofilm. The results showed that the Chl-based PDI increased the visual quality and the content of chlorophyll, VC, total soluble solids, and SOD activity and decreased the occurrence of leaf yellowing, populations of inoculum bacterial, and POD activity. Additionally, the Chl-based PDI reduced the amount of biofilm and inhibited the production of bacterial extracellular polysaccharides and extracellular proteins. The experimental data supported the view that a new generation of photoactive chlorophyllin with 405 nm light could effectively preserve fresh-cut pakchoi in a non-thermal way, which is in line with the requirements of clean and green technology. Thus, PDI has the potential to be used as an antimicrobial tool for treating fresh-cut fruits and vegetables or other food products. However, it also has limitations; for example, it cannot effectively sterilize areas that cannot be reached by light. The feasibility of its application in practical production needs to be investigated in the future.

## Figures and Tables

**Table 1:** L* value of samples during storage.

	0 d	2 d	4 d	6 d	8 d	10 d	12 d
Control	36.12 ± 2.55 ^Ad^	40.69 ± 3.26 ^Ad^	37.73 ± 2.78 ^Bd^	49.67 ± 2.55 ^Ac^	69.9 ± 3.95 ^Aa^	61.49 ± 3.96 ^Ab^	54.21 ± 16.98 ^Abc^
Light	36.12 ± 2.55 ^Ab^	41.27 ± 1.93 ^Aab^	42.7 ± 1.64 ^Aab^	39.36 ± 3.17 ^Cab^	36.53 ± 3.57 ^Db^	44.64 ± 5.43 ^Ba^	42.62 ± 7.01 ^Aab^
Chl	36.12 ± 2.55 ^Ae^	42.51 ± 1.49 ^Abc^	38.02 ± 2.23 ^Bde^	44.86 ± 1.73 ^Bb^	50.15 ± 3.65 ^Ba^	40.53 ± 3.76 ^Bcd^	50.38 ± 3.71 ^Aa^
Chl+Light	36.12 ± 2.55 ^Acd^	38.92 ± 3.08 ^Ac^	32.99 ± 1.39 ^Cd^	34.12 ± 3.17 ^Dcd^	43.67 ± 3.79 ^Cb^	44.59 ± 5.13 ^Bb^	52.38 ± 4.00 ^Aa^

^A, B, C, D^ Means in the same column with different superscripts are significantly different (*p* < 0.05). ^a, b, c, d^ Means in the same row with different superscripts are significantly different (*p* < 0.05).

**Table 2:** The a* value of samples during storage.

	0 d	2 d	4 d	6 d	8 d	10 d	12 d
Control	−17.99 ± 2.76 ^Ac^	−19.69 ± 2.47 ^Ac^	−18.87 ± 0.64 ^Bc^	−17.69 ± 1.75 ^Ac^	−7.85 ± 3.78 ^Ab^	4.78 ± 2.54 ^Aa^	7.73 ± 2.77 ^Aa^
Light	−17.99 ± 2.76 ^Ac^	−19.31 ± 0.99 ^Ac^	−19.01 ± 0.78 ^Bc^	−18.05 ± 1.00 ^Ac^	−15.77 ± 1.17 ^Bc^	−10.76 ± 4.05 ^Cb^	−7.56 ± 3.52 ^Ca^
Chl	−17.99 ± 2.76 ^Acd^	−18.27 ± 1.10 ^Acd^	−18.69 ± 0.88 ^Bcd^	−19.91 ± 0.94 ^Bd^	−15.10 ± 1.18 ^Bc^	−4.72 ± 4.64 ^Bb^	3.78 ± 2.34 ^Ba^
Chl+Light	−17.99 ± 2.76 ^Aa^	−18.81 ± 1.85 ^Aa^	−17.02 ± 0.57 ^Aa^	−17.17 ± 1.42 ^Aa^	−18.86 ± 0.54 ^Ca^	−17.3 ± 1.38 ^Da^	−16.79 ± 2.20 ^Da^

^A, B, C, D^ Means in the same column with different superscripts are significantly different (*p* < 0.05). ^a, b, c, d^ Means in the same row with different superscripts are significantly different (*p* < 0.05).

**Table 3:** The b* value of samples during storage.

	0 d	2 d	4 d	6 d	8 d	10 d	12 d
Control	34.37 ± 1.51 ^Ac^	34.57 ± 2.95 ^Ac^	35.27 ± 2.20 ^Bc^	44.61 ± 2.69 ^Ab^	65.61 ± 4.07 ^Aa^	50.60 ± 2.87 ^Ab^	47.76 ± 15.00 ^Ab^
Light	34.37 ± 1.51 ^Aa^	35.39 ± 3.77 ^Aa^	41.31 ± 2.08 ^Aa^	34.17 ± 1.50 ^Ca^	35.28 ± 3.02 ^Ca^	39.79 ± 8.67 ^Ba^	34.65 ± 5.32 ^Ba^
Chl	34.37 ± 1.51 ^Ab^	35.23 ± 3.68 ^Ab^	37.26 ± 1.40 ^Bb^	38.05 ± 2.69 ^Bb^	44.62 ± 5.00 ^Ba^	34.87 ± 4.67 ^Bb^	37.88 ± 3.63 ^ABb^
Chl+Light	34.37 ± 1.51 ^Ac^	34.54 ± 2.20 ^Ac^	32.49 ± 1.78 ^Cc^	30.77 ± 1.63 ^Dc^	39.22 ± 1.45 ^Cb^	42.11 ± 4.13 ^Bb^	48.56 ± 3.53 ^Aa^

^A, B, C, D^ Means in the same column with different superscripts are significantly different (*p* < 0.05). ^a, b, c, d^ Means in the same row with different superscripts are significantly different (*p* < 0.05).

## Data Availability

Data are contained within the article.
